# Acute Flaccid Myelitis Among Children — Washington, September**–**November 2016

**DOI:** 10.15585/mmwr.mm6631a2

**Published:** 2017-08-11

**Authors:** Jesse Bonwitt, Amy Poel, Chas DeBolt, Elysia Gonzales, Adriana Lopez, Janell Routh, Krista Rietberg, Natalie Linton, James Reggin, James Sejvar, Scott Lindquist, Catherine Otten

**Affiliations:** ^1^Epidemic Intelligence Service, CDC; ^2^Office of Communicable Disease Epidemiology, Washington State Department of Health; ^3^Public Health—Seattle & King County, Seattle, Washington; ^4^National Center for Immunization and Respiratory Diseases, CDC; ^5^Providence Child Neurology, Providence Sacred Heart Medical Center & Children's Hospital, Spokane, Washington; ^6^National Center for Emerging and Zoonotic Infectious Diseases, CDC; ^7^Pediatric Neurology, Seattle Children’s Hospital, Washington.

In October 2016, Seattle Children’s Hospital notified the Washington State Department of Health (DOH) and CDC of a cluster of acute onset of limb weakness in children aged ≤14 years. All patients had distinctive spinal lesions largely restricted to gray matter detected by magnetic resonance imaging (MRI), consistent with acute flaccid myelitis (AFM). On November 3, DOH issued a health advisory to local health jurisdictions requesting that health care providers report similar cases. By January 24, 2017, DOH and CDC had confirmed 10 cases of AFM and excluded two suspected cases among residents of Washington during September–November 2016. Upper respiratory tract, stool, rectal, serum, buccal, and cerebrospinal fluid (CSF) specimens were tested for multiple pathogens. Hypothesis-generating interviews were conducted with patients or their parents to determine commonalities between cases. No common etiology or source of exposure was identified. Polymerase chain reaction (PCR) testing detected enterovirus D68 (EV-D68) in nasopharyngeal swabs of two patients, one of whom also tested positive for adenovirus by PCR, and detected enterovirus A71 (EV-A71) in the stool of a third patient. *Mycoplasma* spp. immunoglobulin M (IgM) titer was elevated in two patients, but both had upper respiratory swabs that tested negative for *Mycoplasma* spp. by PCR. Clinicians should maintain vigilance for AFM and report cases as soon as possible to state or local health departments.

On October 3, 2016, DOH and CDC were notified of a boy aged 7 years who was evaluated for acute onset of limb weakness at Seattle Children’s Hospital. Eight additional patients with limb weakness were reported by the same hospital during that month, including one retrospectively identified patient with onset of weakness on September 14. MRI studies indicated distinctive lesions of the spinal cord largely restricted to gray matter in all nine patients. The clinical presentation and MRI findings among patients were similar to those reported among clusters of cases in other states during 2014 (*1*,*2*). This led to ongoing routine surveillance by DOH in Washington since 2014 and the implementation of a standard case definition for AFM* in 2015. On November 3, DOH issued a health advisory reiterating that local health jurisdictions should report suspected AFM cases.

An AFM case was defined as acute onset of weakness in any limb in persons of any age and either an MRI indicating spinal cord lesions largely restricted to gray matter and spanning ≥1 vertebral segments (confirmed case) or CSF pleocytosis with a white blood cell count >5 cells/mm^3^ (probable case). By January 24, DOH had received patient summary forms^†^ for 12 suspected AFM cases from three health care facilities in Washington. DOH and CDC classified cases on the basis of patient summary forms, MRI reports, and MRI images. During September 2016–January 2017, among 12 suspected AFM cases, 10 were confirmed; two did not meet confirmed or probable case criteria.

Among 10 patients with confirmed AFM, date of onset of neurologic symptoms ranged from September 14 to November 9 (Figure). All patients were hospitalized for treatment of their neurologic illnesses. The median patient age was 6 years (range = 3–14 years); seven patients were male, five were white, one was American Indian/Alaska Native, one was black, and the race of three patients was unknown.

**FIGURE:**
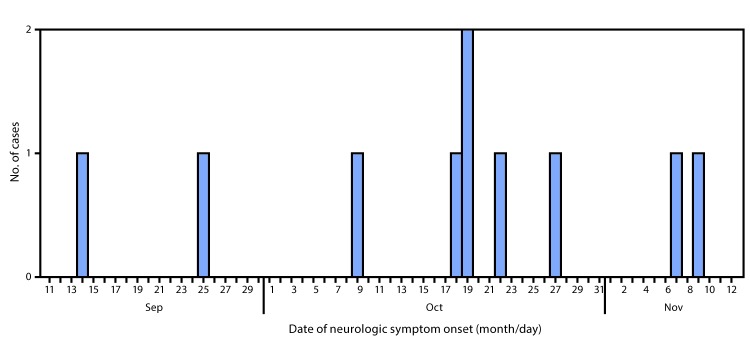
Number of confirmed cases of acute flaccid myelitis (N = 10), by date of onset of neurologic symptoms — Washington, September 14–November 9, 2016

Prodromal respiratory symptoms, gastrointestinal symptoms, or both were reported for eight patients. The median interval from onset of respiratory symptoms to onset of neurologic symptoms was 7 days (range = 1–12 days), and from onset of gastrointestinal symptoms to onset of neurologic symptoms was 2 days (range = 0–12 days). For nine patients, fever was reported in the 4 weeks before the onset of limb weakness. All patients were reported to have been previously healthy, although one patient had an asymptomatic Chiari I malformation. No patient had a reported rash or had previously received immunosuppressing agents. According to patients’ vaccination records, all but one had been vaccinated according to Advisory Committee on Immunization Practices recommendations.^§^ The median interval between receipt of the last vaccination and onset of neurologic symptoms was 1.9 years (range = approximately 2 months–7 years).

All patients initially had acute onset of weakness in one or more limbs. At the peak of neurologic symptoms, eight patients had more than one involved limb, two had three involved limbs, and four patients had all limbs involved. Other neurologic signs included acute neck weakness (one patient), bladder or bowel incontinence (five patients), and cranial nerve dysfunction, including facial weakness or diplopia (three patients). The severity of symptoms in one patient limited parts of the neurologic examination that require patient participation; this patient also required ventilator support. The median duration of hospitalization was 7 days (range = 4–35 days). Four patients received intravenous immunoglobulin and eight patients received intravenous steroids with an oral taper. Seven patients underwent rehabilitation therapy, four as inpatients and three as outpatients. Among nine patients for whom follow-up information was available 1.5–3 months after discharge, five had mild to no residual deficits, three had moderate improvement with residual limb weakness, and one had moderate improvement, but was not ambulatory without assistance. No deaths among confirmed cases occurred.

CSF collection was attempted in all patients; however, contamination with blood rendered one patient’s sample uninterpretable. Seven of nine patients had pleocytosis (median = 163 cells/mm^3^; range = 13–395 cells/mm^3^; reference = 0–5 cells/mm^3^). CSF protein range was 19–99 mg/dL (median = 57.5 mg/dL; reference <40 mg/dL). Nine patients received an MRI of the full spinal cord, and one patient received an MRI of the cervical and upper thoracic region. All patients received a brain MRI. All patients had lesions at the cervical cord level; nine patients had thoracic cord lesions and five patients had lesions at the conus medullaris level (termination of the spinal cord at approximately lumbar [L1/L2]). No MRI reports noted enhancement of the cauda equina (nerve roots descending below the end of the termination of the spinal cord) in any patient. Two patients had supratentorial (the region of the brain containing the cerebrum) lesions, one had subcortical lesions, and three had brainstem lesions. Lesions were predominantly confined to the gray matter in nine patients and to the gray and white matter in one patient. Lesions in two patients displayed enhancement with contrast media, which was used in all patients.

Hypothesis-generating interviews were conducted with seven patients or their parents; three patients or their families declined to be interviewed. Questions covered all activities undertaken 2 months before onset of prodromal symptoms, including contact with sick persons, travel within and outside the United States, and exposures to environmental sources. Household members of four patients reported upper respiratory symptoms during the patient’s prodromal illness. No other household members developed AFM. No patient had traveled outside four states (California, Idaho, Oregon, and Washington). Six patients had participated in open freshwater activities (lakes and rivers), but these took place at separate locations. The patients were residents of six counties in Washington; two patients residing outside of the Seattle metropolitan area lived within 11 miles of each other, but otherwise no spatial clustering was observed. No common environmental exposures were identified.

Specimens were available for all patients, including upper respiratory tract (eight patients), CSF (10), stool or rectal swab (eight), buccal swab (one), and serum (10). Specimens were tested at hospital laboratories in Washington and at CDC’s Picornavirus Laboratory for multiple pathogens, including adenovirus, cytomegalovirus, enteroviruses (including poliovirus), Epstein-Barr virus, herpes simplex virus, human herpes virus 6, influenza, parechoviruses, varicella zoster virus, West Nile virus, and fecal and respiratory bacteria.

EV-D68 was detected by PCR testing in nasopharyngeal swabs of two patients, one of whom also tested positive for adenovirus by PCR. EV-A71 was detected by PCR testing in the stool of a third patient. PCR testing of the CSF from the patient with EV-A71 was also indeterminate for Epstein-Barr virus and human herpes virus 6. Two patients had elevated *Mycoplasma* spp. IgM titers; their IgG titers were within normal range and results of testing upper respiratory swabs were negative for *Mycoplasma* spp. by PCR. Test results for all specimens were negative for poliovirus.

## Discussion

Among a cluster of 10 cases of AFM among children in Washington during September–November 2016, no common etiology or source of exposure was identified. During the preceding year (August 2015–August 2016), no confirmed cases and only one probable case were reported in Washington, consistent with the limited number of AFM cases reported nationally during that period (*3*). In 2014, only two AFM cases were reported in Washington and 120 cases nationally (*4*). The demographic characteristics and clinical presentation of patients in this cluster are similar to those of previously reported cases (*1*,*2*,*4*,*5*). However, among patients in previous clusters for whom follow-up information was available (*2*,*4*,*5*), a larger proportion required ventilator support, reported persistent motor deficits, or were transferred to a rehabilitation facility.

EV-A71 and EV-D68, which were identified in three patients in this cluster, have been associated with outbreaks of neurologic disease (*6*,*7*). However, as in previously reported clusters of AFM, no pathogen was consistently isolated from all specimens tested (*1*,*2*,*8*). The prodrome of fever and respiratory or gastrointestinal symptoms, combined with clinical outcomes consistent with reported cases of neurologic disease from enterovirus and other neurotropic virus infection, suggest that the etiology of these AFM cases is infectious (*4*,*9*). Another possible etiology might be a postinfectious phenomenon, in which the viral infection leads to a delayed immune response and for which laboratory evidence of the involved pathogen might be lacking at the time of weakness onset (*9*). This underscores the importance of timely reporting of cases and expanded AFM testing to include both infectious and noninfectious causes.

The findings in this report are subject to at least two limitations. First, because reporting of AFM is voluntary, incidence in the United States is unknown. Second, because of the clinical similarity between AFM and other neurologic conditions such as idiopathic transverse myelitis and acute inflammatory demyelinating polyneuropathy subtype of Guillain-Barré syndrome, cases might be misdiagnosed and not reported to state and local health departments. AFM typically leads to chronically depressed reflexes, and sensory findings are not typically as discrete as in transverse myelitis, or progressively ascending as in acute inflammatory demyelinating polyneuropathy. AFM lesions indicated on an MRI are more often confined to the gray matter than lesions associated with transverse myelitis, and can also include nerve root enhancement and cranial nerve involvement (*5*,*10*).

Clinicians, specifically pediatric neurologists, should maintain vigilance for AFM. They are encouraged to report cases as soon as possible to state or local health departments to add to information regarding clinical signs, severity, and illness prognosis.

**Summary**

**What is already known about this topic?**

Acute flaccid myelitis (AFM) is a neurologic condition with newly standardized clinical criteria that aid in its recognition. AFM is characterized by acute onset of flaccid limb weakness and lesions in the gray matter of the spinal cord evident on magnetic resonance imaging. Investigation of previously reported clusters did not identify a specific etiology, although during 2014, a temporal association between clusters of AFM and increased incidence of enterovirus-D68 (EV-D68) infections was reported. Because reporting is voluntary, the range of clinical signs, severity, and incidence in the United States is difficult to determine.

**What is added by the report?**

During September–November 2016, 10 confirmed cases of AFM were reported in Washington. No common etiology or source of exposure was identified. Enterovirus-A71 was detected in one patient and EV-D68 in two patients, one of whom also tested positive for adenovirus. *Mycoplasma* spp. immunoglobulin M titer was detected in two patients, but polymerase chain reaction testing of an upper respiratory swab was negative in both.

**What are the implications for public health practice?**

Clinicians should remain vigilant for AFM and report cases to state or local health departments as soon as possible. Timely collection of specimens for laboratory testing and expansion of testing to include infectious and noninfectious causes might help uncover a common etiology within a cluster.

## Footnotes

* http://c.ymcdn.com/sites/www.cste.org/resource/resmgr/2015PS/2015PSFinal/15-ID-01.pdf.

† https://www.cdc.gov/acute-flaccid-myelitis/hcp/data.html.

§ https://www.cdc.gov/vaccines/schedules/hcp/child-adolescent.html.
